# Three dimensional movement analysis of maxillary impacted canine using TADs: a pilot study

**DOI:** 10.1186/s13005-020-00252-0

**Published:** 2021-01-15

**Authors:** Marco Migliorati, Lucia Cevidanes, Giordana Sinfonico, Sara Drago, Domenico Dalessandri, Gaetano Isola, Armando Silvestrini Biavati

**Affiliations:** 1 Orthodontics Department, School of Dentistry University of Genova largo Rosanna Benzi, 10 16132 Genoa, Italy; 2 Department of Orthodontics and Pediatric Dentistry, University of Michigan, School of Dentistry, Ann Arbor, USA; 3 Private Practice, Genoa, Italy; 4 Department Of Orthodontics, School of Dentistry, University of Brescia, Brescia, Italy; 5 Department of General Surgery and Surgical-Medical Specialties, University of Catania, Catania, Italy

**Keywords:** 3D imaging, Impacted canines, Anchorage

## Abstract

**Background:**

The aim of the present study was to compare two different anchorage systems efficiency to disinclude impacted maxillary canines using as evaluation tool superimposed Cone Beam Computed Tomography (CBCTs).

**Methods:**

The study has been conducted with two parallel groups with an allocation ratio of 1:1. Group test received treatment using as anchorage a miniscrew, control group was treated using an anchorage unit a trans palatal arch (TPA). Both groups received a calibrated traction force of 50 g. CBCT before treatment and 3 months after traction were superimposed and canine tip and root movement were evaluated in mm/month ratio.

**Results:**

No differences were observed between groups for apex displacement, tip displacement and observation timespan. Twenty-two patients (12 female, 10 male, mean age:13.4 years) undergoing orthodontic treatment for impacted maxillary canines were recruited for this study. No differences were observed between groups for apex displacement, tip displacement and observation timespan.

**Conclusions:**

The present pilot study provided no evidence that indirect anchorage on miniscrews could make canine disimpaction faster than anchorage on a TPA.

An apex root movement of 0.4–0.8 mm per month was found, while average canine tip movement ranged between 1.08 mm and 1.96 mm per month.

No miniscrews failures were observed.

**Trial registration:**

The study reports the preliminary results of the randomized clinical trial registered at www.register.clinicaltrials.gov (registration number: NCT01717417).

## Background

Maxillary canines are the second-most frequently impacted teeth in the dental arch after the third molars with prevalence from 1 to 3%. Most of the impactions are palatal (85%), whereas 15% are labial [1]. The absence of the permanent canine in the arch, after the normal eruption timing, leads clinicians to suspect canine impaction and that has to be confirmed by a clinical evaluation of the patient and a radiographic assessment. The approaches to the management of impacted canines are many, but the preferred approach typically involves surgical exposure and guided orthodontic eruption [2].

Guided orthodontic eruption of palatally impacted canines may lead to prolonged treatment, and potential negative consequences, such as the propensity for greater root resorption and poor patient compliance [3].

The intraosseous orthodontic biomechanics for traction of impacted canines can be achieved in several ways, but the anchorage method plays a crucial role in the success and guided control of the direction of canine eruption. A transpalatal arch (TPA) is ordinarily used to stabilize the upper arch during canine traction and eruption. However, the anchorage unit may be also transferred to the mandibular arch [4]. Temporary Anchorage Devices (TADs), and cantilevers with a Ttitanium Molybdenum Alloy (TMA) sectional represent alternative methods for anchoring the system: a TAD is a mini screw temporarily fixed to the bone for the purpose of enhancing orthodontic movement, either by supporting the reactive unit (indirect anchorage) or by obviating the need for it (direct anchorage), and is subsequently removed after use [2]. A number of clinical studies and reviews have investigated the stability of TADs for acting as stable anchor units [5–8]. In a recent study investigating anchorage loss when using a conventional TPA in comparison to a mini-screw implant, minimal mesial movement of the maxillary first molars was observed when mini-screw implants were placed and passively engaged prior to leveling and aligning. On the other side, approximately 2.5 mm of mesial movement of the first molars was observed when using a TPA. The loss of anchorage was evaluated during maxillary canine retraction with fixed appliances [9].

New possibilities for the evaluation of canine impaction treatment effects have been introduced by CBCT superimposition techniques that have allowed a novel and accurate approach in the quantification of tooth displacement [10–13]. These techniques made it possible not only to perform a 3D evaluation, but also to observe the path of a tooth impacted in the bone layer.

Hence an important clinical question arose regarding the different types of anchorage for canine traction: could a difference be observed during the intraosseous traction of the maxillary canine too? In particular, could the different type of anchorage determine a difference in the speed, direction and amount of canine movement?

To our knowledge, previous studies have not proposed a comparison between the effects of anchorage on a TPA and indirect anchorage on a miniscrew for the impacted canine displacement. The null hypothesis was that there is no difference in traction speed, direction and amount between the impacted canines anchored on a TPA and the impacted canines indirectly anchored on a miniscrew.

## Methods

### Study design

The present study reports the preliminary results of the randomized clinical trial registered at www.register.clinicaltrials.gov with registration number XXXXXXXXX.

### Trial design

The study has been conducted with two parallel groups with an allocation ratio of 1:1.

### Participants

Inclusion criteria for the patients were the following:

- presence of one or two impacted maxillary canine requiring surgical exposure and orthodontic treatment.

Exclusion Criteria:

- permanent teeth extraction-based treatment
- current or previous orthodontic treatment in the last 12 months
- current systemic disease
- current antibiotic or anti-inflammatory therapy that could possibly compromise the results.

### Interventions

**Fig. 1:**
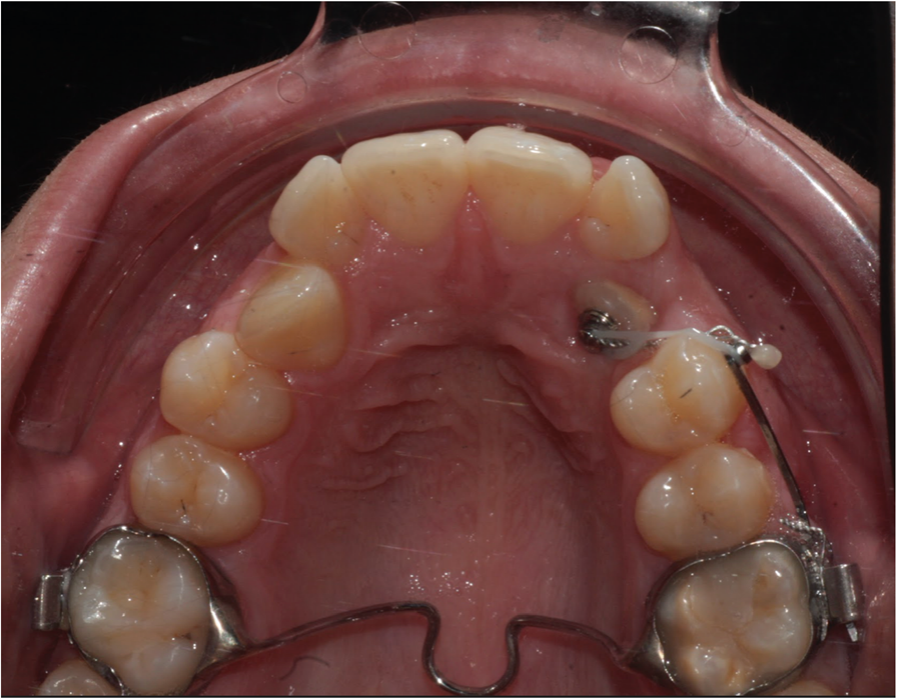
group 1 intervention: trans palatal arch anchorage

**Fig. 2:**
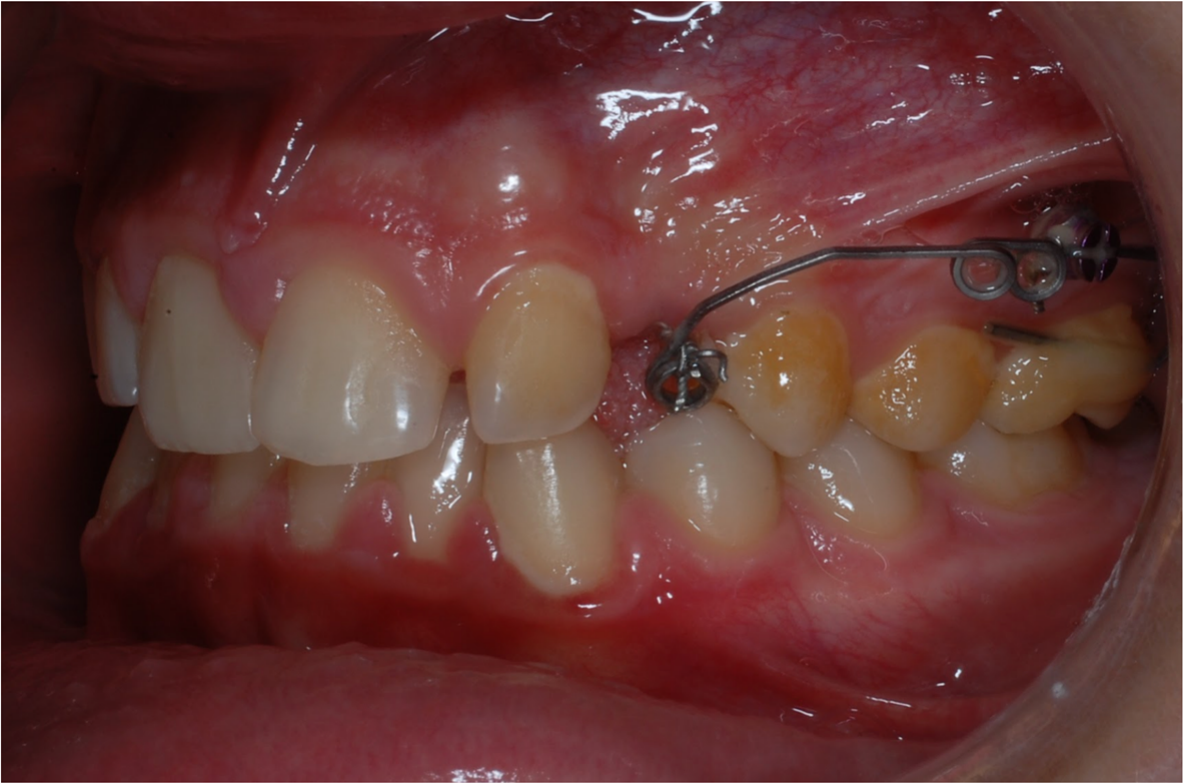
group 2 intervention: miniscrew indirect anchorage

Two kind of interventions were planned: the first group of patients received a TPA as anchorage unit for canine traction (Fig. 1); in the second group intervention was held with a TAD as anchorage unit (Fig. 2).

In the TAD group, an 8 mm long miniscrew (Orthoeasy, Forestadent, Pforzheim, Germany) was used as anchorage. In both groups the approach to solve the impaction was “canine first”: no anchorage preparation was performed, besides the TPA or the miniscrew. The biomechanics included a Beta-titianium cantilever applying a force of 50–60 g measured with a pen gauge; biomechanics varied among the patients including extrusive, and distalizing vector force. The miniscrews insertion sites varied depending on the impacted canine position, as well as the cantilever in the TPA group. The day of the surgical exposure of the canine was coincident with the beginning of traction. Close surgical intervention technique was performed for all included patients.

### Imagine methods

All patients agreed in having 2 CBCTs in two different time points of treatment as it was described in the experimental protocol approved by the ethical committee and in the patient’s consensus form.

The first scan was taken before the surgical exposure of the canine and beginning of traction (T0), and the second one about 3 months after (108 days in the test group, 105 in the control group) (T1).

### Image acquisition

Due to variations in the CBCT image acquisition protocol in this study scans, the “Downsize” tool in Slicer was utilized to standardize the image resolution and avoid any heterogeneity of the imaging data. All scans were reformatted to a 0.5 mm^3^ voxel the original scans of 0.4 mm^3^ voxel size using SlicerCMF version 4.0 (https://sites.google.com/a/umich.edu/dentistry-image-computing/) to standardize the scan resolution and decrease the computational power and time for image registration.

### Creation of the virtual model

The first step in image processing was to export the scans in Digital Imaging and Communications in Medicine (DICOM) format and then to convert them into GIPL format for image de-identification.

From the cross-sections of the volumetric data set, virtual three-dimensional models from T0 and T1 scans were created, using ITK-SNAP open-source software. This process, called segmentation, required outlining the shape of the dental arches visible in the slices, setting up a threshold of the tissues density in order to select the anatomical structure of interest.

**Fig. 3:**
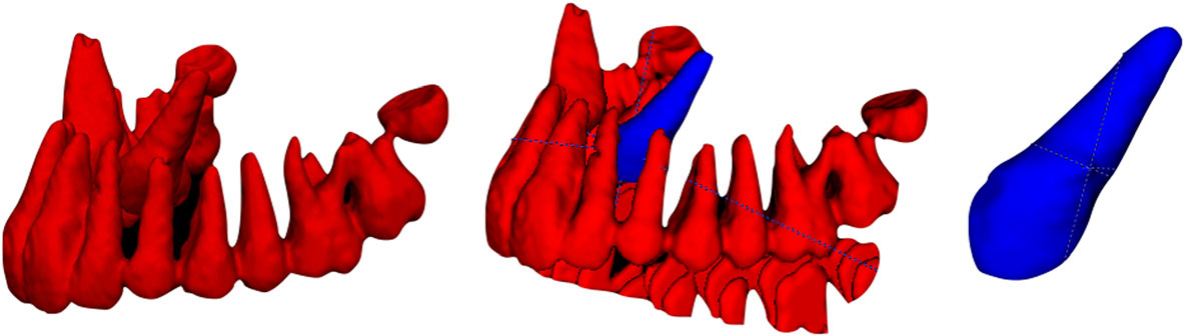
arch 3D model after segmentation

Hence the 3D model of the canine could be isolated from the *rest* of the dental arch (Fig. 3).

### Registration

The first step in the registration process was to determine which structure would be used as a stable reference following the maxillary regional registration methods validated by Ruellas et al. [14]. As the timespan was very short for a significant skeletal growth, and the treatment had only dental effects, the maxillary bone was considered a good structure for reference.

The registration procedure does not depend on the precision of the 3D surface models, but actually compares voxel by voxel of gray level CBCTs images, and calculates the rotation and translation parameters between the 2 time point images.

This is a fully automated process, that is simplified by a primary manual overlap of the two CBCTs using CMF registration module in SlicerCMF (https://sites.google.com/a/umich.edu/dentistry-image-computing/).

Once both images from different time points are registered, they share the same coordinate system.

### Overlay of the 3D models and quantitative measuraments

**Fig. 4:**
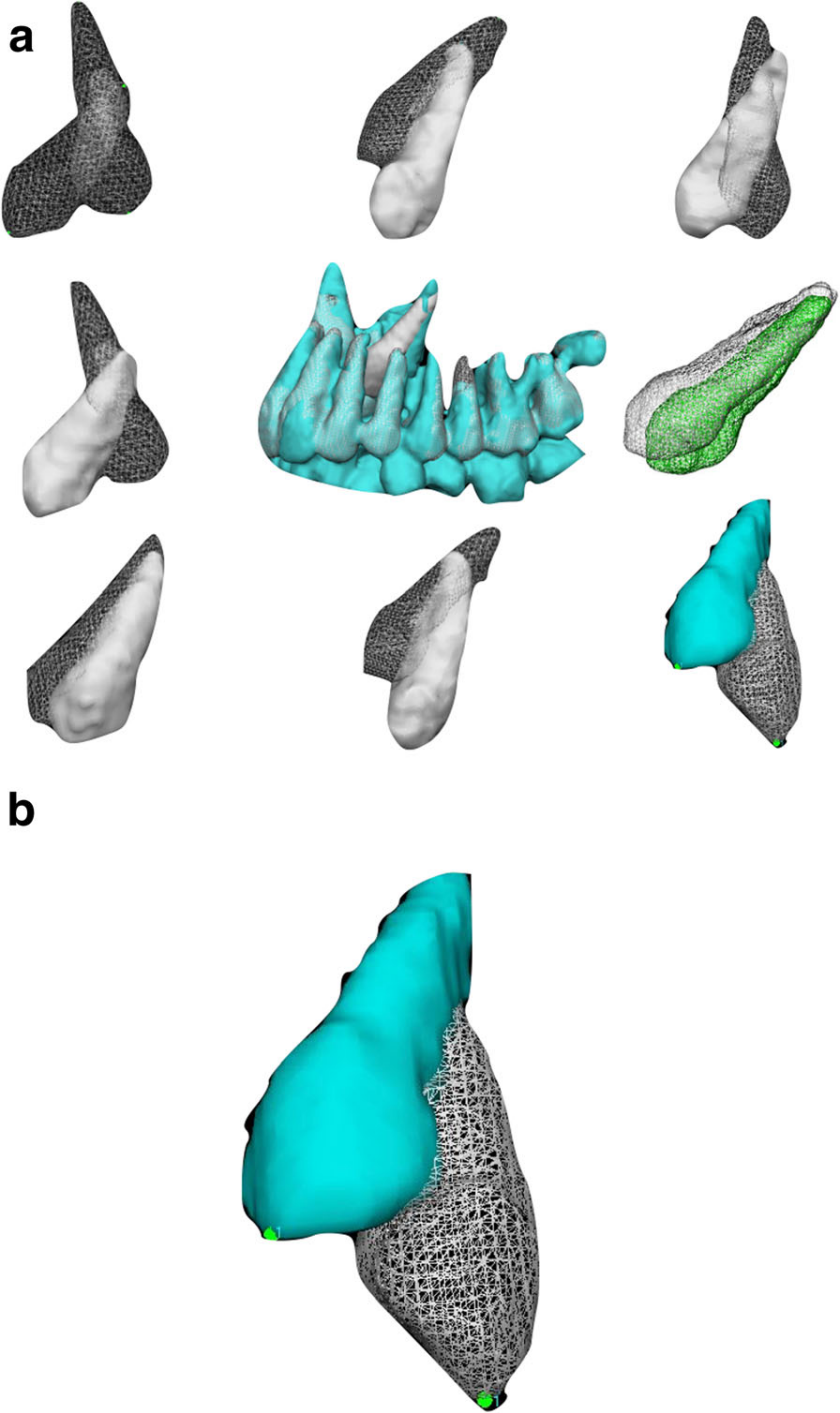
**a** t0 and t1 3D models superimposed after registration of different treated canines. **b** reference tip point for linear measurements

The next step included the use of VAM software (VAM v. 3.7.6, Canfield 113 Scientific Inc., Fairfield, NJ) for overlaying the registered 3D models, that allowed to evaluate the displacement of the canine and to measure the distance between the tip of the cusp of the canine resulting from the CBCT at T0 and that at T1, as well as for the apex. The software allows the selection of two points and calculates distance in mm between two points (Fig 3, 4a, b).

### Color maps

**Fig. 5:**
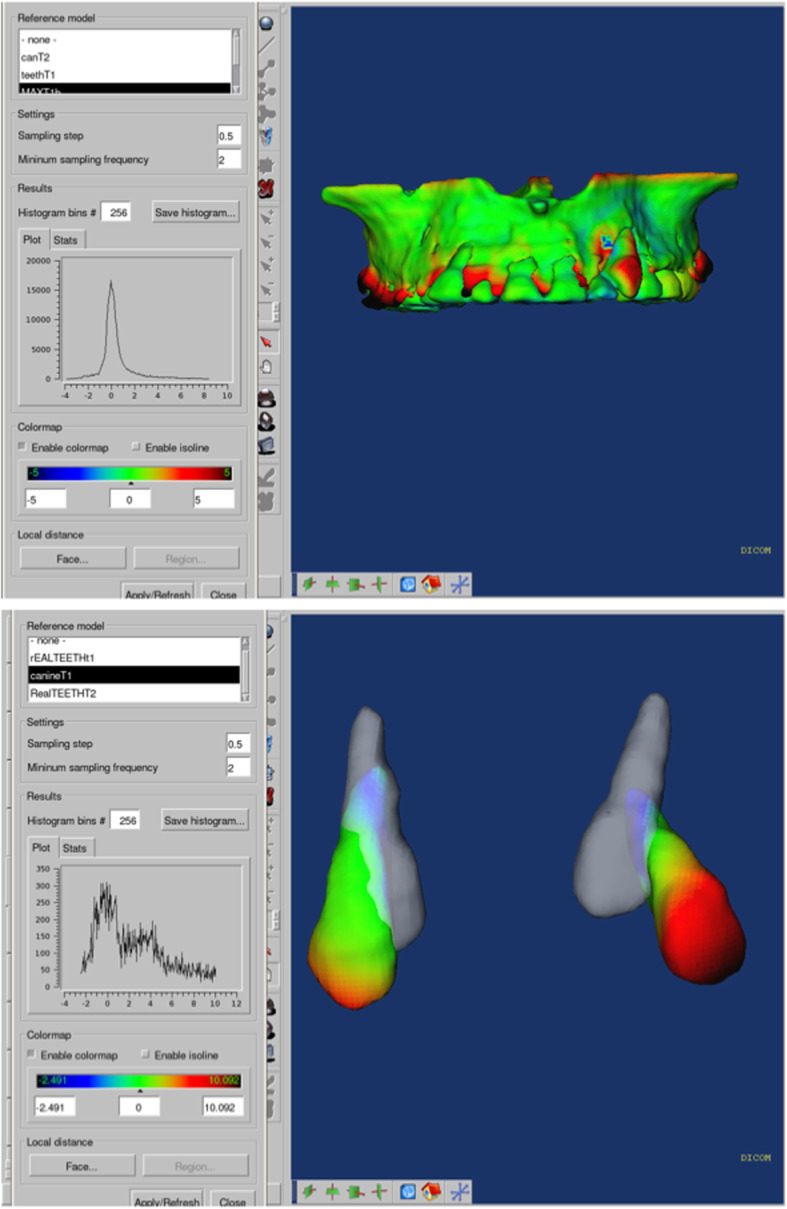
Color Maps

Gerig et al. proposed the use of color maps generated from closest-point distances between the surfaces [15]. The CMF tool calculates thousands of color-coded surface distances in millimeters between 3D models surface triangles at two different time points. The color maps indicate inward (blue) or outward (red) displacement between overlaid structures. An absence of changes is indicated by the green color. In this study, color-coded maps were utilized just for visualization and qualitative assesments, but not to measure canine movement (Fig. 5).

### Outcomes

The primary outcome measurement was the canine speed of movement evaluated using a voxel based superimposition of two consecutive CBCTs. The CBCTS were acquired at baseline and 3 months after starting treatment for both groups. Once the linear displacement of the canine was measured, this was divided by the observation period in weeks to obtain the ratio of mm/week movement.

### Randomization

The randomization list was generated by a customized software, allowing a random list with an allocation ratio 1:1.

### Blinding

All the statistical analysis was blindly performed in regards of patient’s group origin.

### Measurement repeatability

All the measurements were repeated by the same operator 1 month after the first examination and intraclass correlation coefficient was calculated both for the apex and canine tip. Intraclass Correlation Coefficient (ICC) values were 0.87 and 0.88 for canine tip and canine root apex respectively.

### Statistical analysis

Descriptive statistics are expressed as median and interquartile ranges. The data were tested for normality using the Shapiro-Wilk test. The nonparametric Spearman’s rank correlation test was used to evaluate the dependence among the measured characteristics.

The nonparametric Mann-Whitney U test was used to evaluate differences between groups. Differences with a *p*-value less than 0.01 were selected as significant and data were acquired and analyzed using R v3.4.4 software environment [16].

## Results

Twenty-two patients (12 female, 10 male, mean age: 13.4 years) undergoing orthodontic treatment for impacted maxillary canines (both labial and palatal) were recruited for this study.

Patients were either treated with a TAD or TPA following the generated randomization list.

**Fig. 6:**
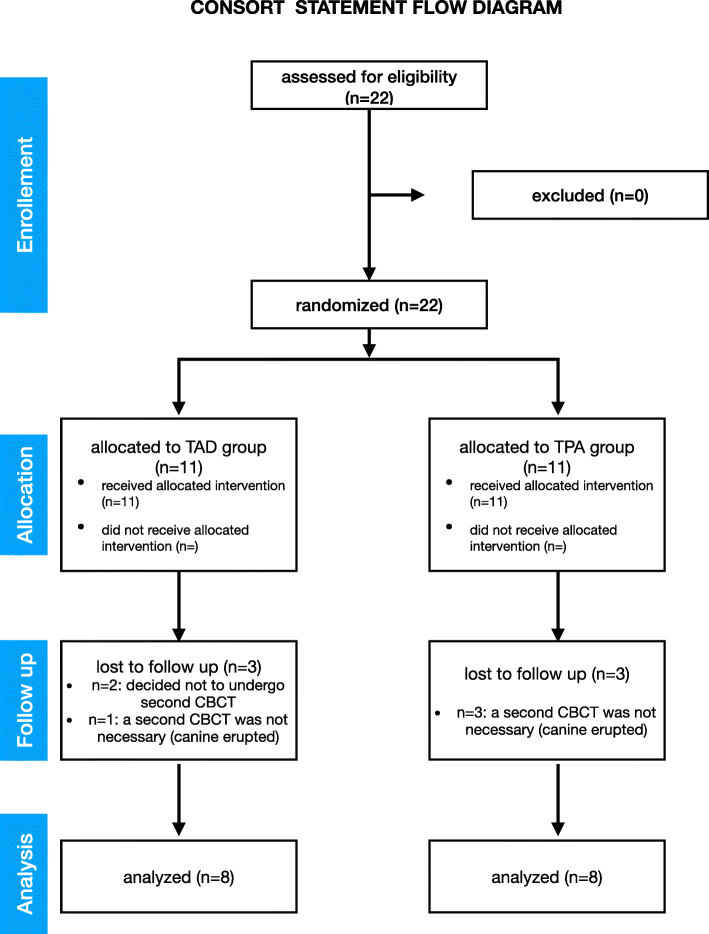
CONSORT study flowchart diagram

TAD and TPA group included 11 patients each. During the observation period, 2 patients decided not to undergo a second CBCT (TAD group) and for other 4 patients a second CBCT was not requested because the canine was already erupted (Fig. 6); 7 out of 16 final sample examined showed buccally displaced canines.

The trial was registered on October 2012 and the recruitment started thereafter.

**Fig. 7:**
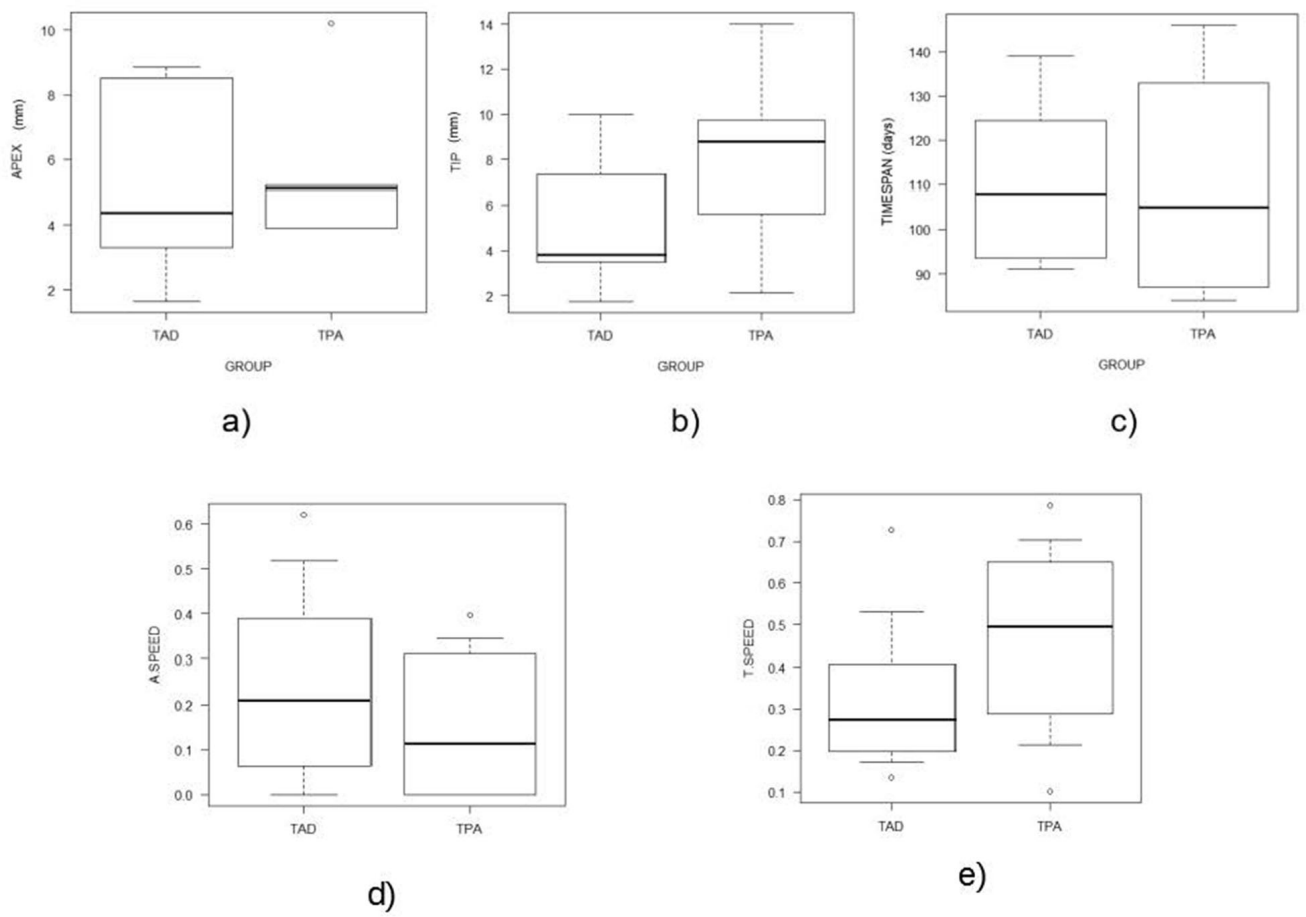
medians and interquartile ranges graph of: a) apex displacement b) tip displacement c) observation timespan d) apex speed e) tip speed

No differences were observed between groups for apex displacement, tip displacement and observation timespan (Table 1, Fig. 7).

**Table 1:** Differences between groups with regard to canine displacement, observation timespan and canine speed (Mann Whitney test values, * *p*< 0.05)

	Minimum	25%	Median	75%	Maximum	***p***-value
**Apex displacement**						
Group TAD	1.65	3.46	4.37	7.59	8.84	0.41
Group TPA	3.90	3.90	5.10	5.20	10.20	
**Tip displacement**						
Group TAD	1.74	3.50	3.81	6.58	9.97	0.189
Group TPA	2.12	5.60	8.80	9.75	14.00	
**Timespan (days)**						
Group TAD	91	94.75	108	121.75	139	0.799
Group TPA	84	88.50	105	126.50	146	
**Apex speed (mm per week)**						
Group TAD	0	0.09	0.21	0.35	0.62	0.67
Group TPA	0	0	0.11	0.27	0.40	
**Tip speed (mm per week)**						
Group TAD	0.13	0.20	0.27	0.38	0.73	0.37
Group TPA	0.10	0.38	0.49	0.58	0.79

**Fig. 8:**
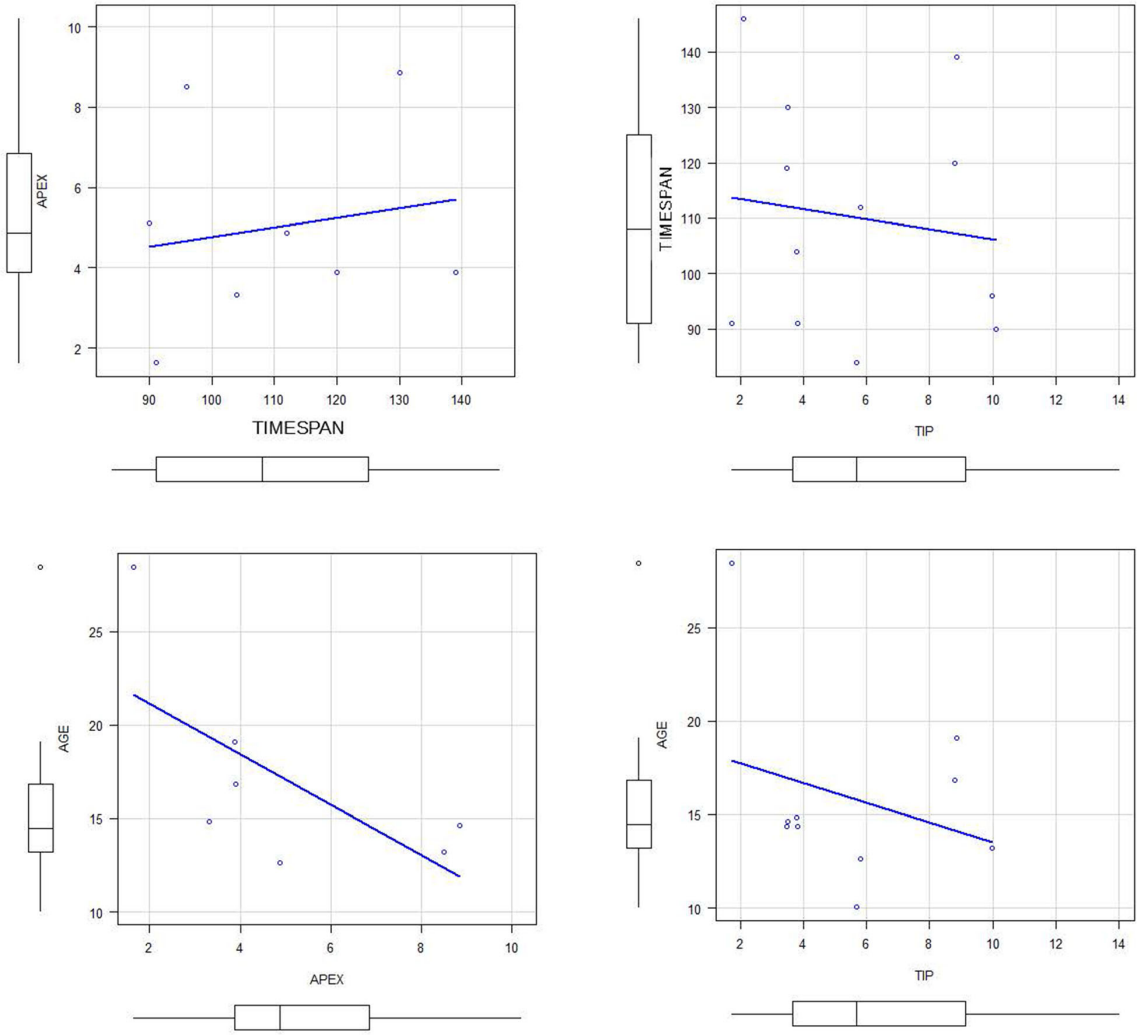
graphical representation of data distribution and correlation line (not significant)

No correlations were found between apex displacement and observation timespan, or patients age. No correlations were found between tip displacement and observation timespan, or patients age (Table 2, Fig. 8).

**Table 2:** Spearman’s rank correlation coefficients for displacement parameters and time or age

		TIMESPAN	AGE
APEX	Correlation	0.0952	−0.75
	P-value	0.84	0.0663
TIP	Correlation	−0.221	−0.224
	P-value	0.491	0.537

## Discussion

### Limits of the study

The main source of potential biases relied on the patients adherence to the appointments, in particular this could affect a constant reactivation of the canine traction and, on the other hand, a longer observation period; in some cases, the second CBCT exam was taken 1.5–2 months later than the requested date, leading to a longer observation timespan. However, the second time point distribution was not significantly different between groups (Table 2) and a comparison was possible.

Moreover, no correlation was found between the canine displacement and the inter-exams time interval. In future studies, the observation of palatally displaced canines and buccally impacted canines in separate groups may aid a more detailed interpretation of tooth movement related to the differences in anatomy and biomechanics indications.

### Generalizability and interpretation

No significant differences were found between groups as regards apex displacement, whose medians were estimated around 4.4 and 5.1 mm in TAD and TPA group respectively. This does not guarantee that the two treatments had the same effects on apex displacement, but it is likely that differences were small and would be significant only in a larger sample.

The same could hold with regard to tip displacement, except that the difference between groups was much larger (5 mm average greater displacement at the tip for the TPA group) even though not significant, due to large variability is response. The greater average differences between groups in tip compared to apex displacements could be due to remaining canine root development or to an angular component of the movement if one of the groups of canine traction presented a wider circular or curved trajectory. This effect was not demonstrated but could depend on the biomechanics of each technique, as well as density differences in the surrounding bone. A recent study comparing subjects with unilateral and bilateral canine impaction found an increased bone area in the impacted side [17]. Even though the amount of bone surrounding the canine traction path was not measured in this study, it is quite reasonable that bone density and different locations of the impacted canine may affect the canine displacement speed.

Interestingly, the anchorage loss in an important consideration, not for the absolute canine movement, but in terms of treatment efficacy: none of the miniscrews was lost and the force of traction did not affect TADs stability, while in TPA group significant molars tipping was clinically observed. Acquisition of larger field of view CBCT scans would have allowed quantification of molars movement, but in this study protocol, the choice was to reduce the field of CBCT exposure to the canine area only.

Recently, a direct proportionality law has been established between the alignment time of a palatally impacted upper canine and the eruption path length.^11^ For this reason, buccally or palatally displaced canines eruption path length may differ and probably deserve separate analysis. However, in the present pilot study they were almost equally represented in both groups of treatment, as often a TPA may also help buccally and obliquely displaced canines and guide a more favorable eruption path. This study design focused on comparing the treatment modalities rather than the canine location. Further information could be gathered in larger samples by relating the displacement timespan to a three-dimensional assessment of impaction severity [18–20]. Future studies including also analysis of direct digital scans may also provide information regarding differences in gingival thickness that may play a role in determining the final eruption time [21–24].

In summary, the evaluation of the canine movement speed in the present study revealed that the rate of eruption was in average 1.08 and 1.96 mm in 1 month in the TAD and TPA groups respectively measured at the tip of the canine applying always a 50–60 g force. At the root apex showed a 0.84 mm and 0.44 monthly movement (TAD and TPA respectively). It is evident how the tip/apex root ratio movement was related to the original inclusion position of the tooth.

A completion of the study with more patients is required to confirm these preliminary data.

## Conclusions

The present pilot study provided no evidence that indirect anchorage on miniscrews could make canine disimpaction faster than anchorage on a TPA.

An apex root movement of 0.4–0.8 mm per month was found, while average canine tip movement ranged between 1.08 mm and 1.96 mm per month.

No miniscrew failures were observed.

## Data Availability

The dataset supporting the conclusions of this article is included within the article additional files.

## Abbreviations

CBCT: Cone beam computed tomography
TPA: Trans palatal arch
TADs: Temporary Anchorage Devices
TMA: Ttitanium Molybdenum Alloy
DICOM: Digital Imaging and Communications in Medicine
ICC: Intraclass Correlation Coefficient
